# The Swedish MS registry – clinical support tool and scientific resource

**DOI:** 10.1111/ane.12425

**Published:** 2015-06-05

**Authors:** J Hillert, L Stawiarz

**Affiliations:** 1 Department of Clinical Neuroscience, Karolinska InstitutetStockholm, Sweden; 2 Department of Neurology, Karolinska University HospitalStockholm, Sweden

**Keywords:** multiple sclerosis, MS registry, national registry, electronic medical record, graphical medical records, clinical research, outcome measures, EDSS, disease-modifying drugs, multiple sclerosis epidemiology, multiple sclerosis genetics, multiple sclerosis immunology, MRI, PROMs

## Abstract

The Swedish MS registry (SMSreg) is designed to assure quality health care for patients with multiple sclerosis (MS). It has been active since 2001 and web-based since 2004. It runs on government funding only and is used in all Swedish neurology departments. The SMSreg currently includes data on 14,500 of Sweden's estimated 17,500 prevalent patients with MS. One important function of SMSreg, to which participation is voluntary, is to serve as a tool for decision support and to provide an easy overview of the patient information needed at clinical visits. This is its core feature and explains why the majority of Swedish MS specialists contribute data. Another success factor for SMSreg is that entered data can be readily accessed, either through a query function into Excel format or through a set of predesigned tables and diagrams in which parameters can be selected. Recent development includes a portal allowing patients to view a summary of their registered data and to report a set of patient-reported outcomes. SMSreg data have been used in close to 100 published scientific reports. Current projects include an incidence cohort (EIMS), post-marketing cohorts of patients on novel disease-modifying drugs (IMSE), and a prevalence cohort (GEMS). As these studies combine physical sampling and questionnaire data with clinical documentation and possible linkage to other public registries, together they provide an excellent platform for integrated MS research.

## Introduction

The Swedish MS registry (SMSreg) is web-based and an integrated part of Swedish MS care, and is used by all departments of neurology throughout the country. Legally, it is part of the local clinical documentation system. Each night, data from the local databases are merged into a compiled data set that constitutes the national SMSreg. Patients are informed about this merging of data and are free to opt out.

Participation in SMSreg is voluntary also from the neurologist's side. However, after 14 years of existence, it has annually recruited data on close to 1000 new patients and thus reached coverage of almost 80% of the estimated prevalent MS population of Sweden. This remarkable development and its reasons will be described in this paper. Throughout this period, the authors have served as chairman (JH) and technical coordinator and database developer (LS) for SMSreg.

## Early years

MS research has a long tradition in Sweden. In particular, the cohort of incident MS patients originally identified by Tore Broman in Gothenburg in the 1950s has been updated regularly by him and later by Oluf Andersen and his team 1. Therefore, the concept of collecting long-term clinical information on patients with MS had already been established when disease-modifying drugs (DMDs) to MS appeared in the mid-1990s. Accordingly, several additional nodes of collection of clinical MS data emerged, some prompted by a perceived need to keep track of patients such as in Uppsala, or by an interest in epidemiology in Umeå and Linköping, or in neuroimmunology and MS genetics in Stockholm. In the late 1990s, discussions began on whether a common set of definitions and assessed parameters could be agreed upon. Joint software, named the Interactive Database for Multiple Sclerosis (IDMS), was developed and implemented at the Karolinska University Hospital. IDMS was demonstrated at the 14th ECTRIMS meeting in Stockholm in 1998. Finally, in 2000 an agreement was met and a joint application was submitted to set up a ‘quality registry’ for MS. Following approval, 2001 became the first official year of SMSreg. To date, SMSreg has been fully financed via the governmental grant system available for national ‘quality registries’ in Sweden. The annual budget for running SMSreg together with the other registries in the Swedish Neuro Registry (see below) is € 600,000.

## Consensus and growth

One of the first expressed purposes of SMSreg was to collect data to assess the long-term effectiveness of MS DMDs. Therefore, the core data set agreed upon contained information not only on demographics, diagnostics, and course but also on DMD treatment. As long-term outcome was the first interest, an emphasis was given to disability as assessed by EDSS and conversion to secondary progression rather than to relapse rates. In addition, to allow comparability of patient groups, we agreed to record the prognostic characteristics of the presenting MS attack as identified by the Gothenburg group 2.

With several cohorts already available to be entered into the registry, each representing a valuable scientific asset, a condition for merging of data became the governmental aspect of how to distribute the benefits of analyzing data. Thus, we agreed to set up a registry research board with the task of assessing governmental aspects not usually covered by ethics boards, such as avoidance of parallel research on the same data. In addition, to ensure a professional engagement as well as influence, the registry board took the initiative to launch a professional Swedish MS Society (SMSS), which was founded in 2003. Ever since, the SMSreg board is a sub-committee of the SMSS.

In its first years, SMSreg was based on a client–server solution, thus requiring hardware, software installed on each computer, and local IT support. This was an expensive and economically inefficient model. Therefore, the software provider, Carmona AB, proposed in 2003 that SMSreg should go web-based. This solution was implemented in 2004.

**Figure 1:**
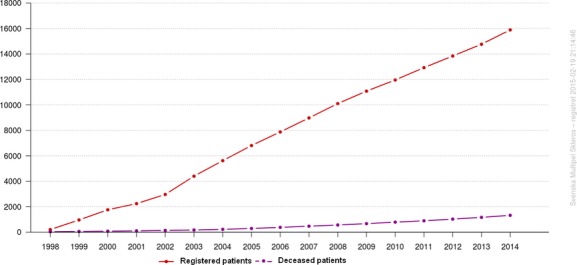
The number of patients registered in SMSreg has increased in a strikingly linear fashion ever since its start in 2001, although participation is voluntary both for patients and clinicians.

As a consequence of pre-existing databases, a consensus on focus, parameters, and governance, and being available on computers in every clinical office, SMSreg began to steadily increase its patient numbers in spite of registration being voluntary (see Fig.1).

## Core data set, variables, and interface

SMSreg is based on an intuitive set of basic parameters, selected through their properties of being clinically relevant. Table1 lists the most important variables including the compulsory ones. A full list of the parameters which it is possible to assign and follow through SMSreg is available at http://www.socialstyrelsen.se/register/registerservice/nationellakvalitetsregister/svenskaneuroregistret.

**Table 1:** The most important parameters of the Swedish MS registry, of which some are mandatory and others recommended or optional, depending on physician's preference or participation in local or national focused follow-up projects such as IMSE (see text). Typically physicians tend to enter data that they find useful for summarizing patient information or for statistics

Module	Variable	Mandatory variable
patient	Registration date	Yes
	Informed consent	Yes
	ID number	Yes
	Date of birth	Yes
	Gender	Yes
	First name	
	Last name	
	Physician in charge	Yes
	Centre/neurological clinic	Yes
	Exclusion date	
	Reason for exclusion (death, migration, own request, other)	
	Date of onset	
	Date of onset approximate (Y/M/D)	
	Fullfilled McDonald's criteria (Y/N)	Yes
	Date of diagnosis	
	Date of diagnosis approximate (Y/M/D)	
	Clinical course (RR, SP, PP, PR)	
	Progression to SP (year)	
clinical visit	Date of visit	Yes
	Physician at visit	Yes
	SF36 (first question)	
	EDSS score	
	Adverse events	Yes
	New bouts since last registered bout (Y/N)	Yes
therapy	Disease Modifying Drug (DMD)	Yes
	Treatment start, date	Yes
	Dose	
	Treatment discontinued, date	Yes
	Reason discontinued	
bout	Bout date	Yes
	Verified by (neurologist, other physician, anamnestic)	Yes
	Corticosteroids treatment	
	Bout type: Isolated ON	
	Bout type: Afferent signs and symptoms only (not ON)	
	Bout type: Only one functional system involved	
	Bout type: Complete remission	
csf	LP date	Yes
	LP performed at	
	OCB (Y/N)	
	IgG index	
	Albumin ratio	
	Number of Mono and PMN leukocytes	
	Intrathecal IgG production (Y/N)	
mri	MRI date	Yes
	MRI exam (diagnostic, follow up)	
	MRI exam of brain (Y/N)	
	T2 lesions (total number)	
	New or enlarged T2 lesions (number)	
	Gd-enhanced lesions in a brain (total number)	
	Comparison with the previous brain MRI, date	
	MRI exam of spinal cord (Y/N)	
	Lesions in spinal cord (Y/N)	
	Spinal cord lesion-load change (increased, decreased, not changed)	
	Gd-enhanced lesions in a spinal cord (total number)	
	Comparison with the previous spinal cord MRI, date	
	Quantitative/volumetric MRI (Y/N)	
	Processing software (SyMRI, FSL, SPM, Freesurfer, other)	
	Brain Parenchymal Fraction (BPF)	
	Normalized total brain volume	
	Normalized total lesion volume in white matter	
msis29	MS Impact Scale (MSIS-29)	
sdmt	Symbol Digit Modalities Test (SDMT)	
msfc	MS Functional Composite (MSFC)	
fss	Fatigue Severity Scale (FSS)	
fsmc	Fatigue Scale for Motor and Cognitive Functions (FSMC)	
eq5d	EQ-5D	
work	Activity/Work capacity	
NAb-lab	Screening for NAb against interferon beta and natalizumab	
laboratory	Lab data (JCV serology, blood lymphocytes)	

**Figure 2:**
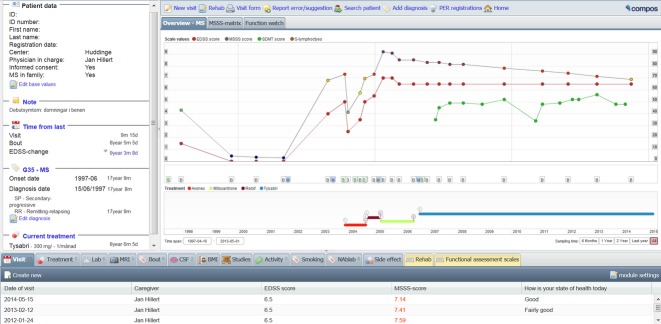
The user interface of the SMSreg, available for each patient, is designed to give a quick overview of relevant clinical information, to facilitate clinical work for the neurologist and MS nurse in relation to clinical visits.

SMSreg is modular, indicating that different sets of data are grouped intuitively. Fig.2 shows the graphical outline of the starting page available for all registered patients. In addition to basic demographics and information on onset of disease, clinical course, and time since important events, the interface is dominated by a graphical representation of the evolution of the patient's EDSS/MSSS above a linear depiction of previous and present DMD treatments on a common time axis. The lower part of the user interface opens access to the different modules in which detailed information on clinical visits, treatments, MS attacks, imaging, and functional tests are gathered.

## Current features and development

Our hypothesis is that usefulness of the registry to those who enter data is the main motivational factor. This is the leading principle in developing SMSreg further. Thus, we have consistently adapted the content of the registry by omitting variables not used while seeking ones that are clinically or scientifically relevant.

### Decision support tool

**Figure 3:**
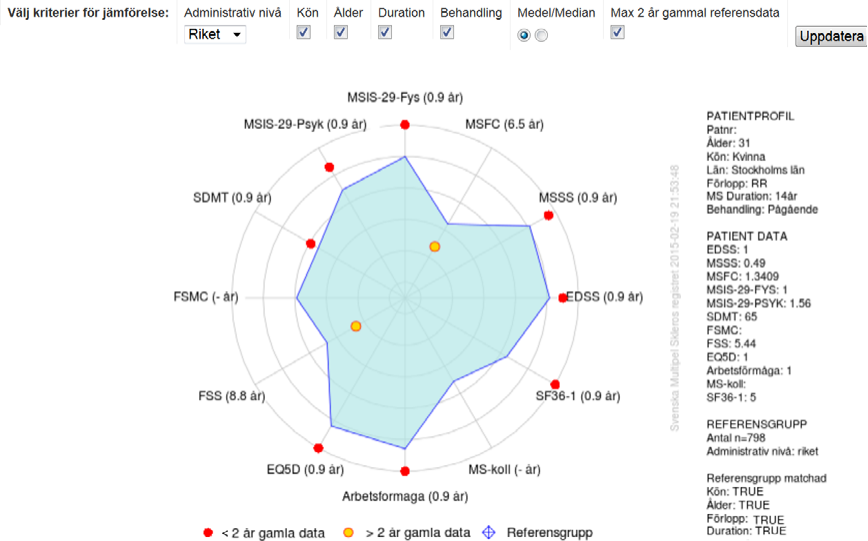
The ‘Function Watch’ radar diagram showing the result of a patient along 12 parameters available in the SMSreg, including the MSIS-29 3, the EQ5D, 4 ‘arbetsförmåga’ (working capacity), and other items listed below. The red points indicate the current performance of the patient, whereas the blue field shows the results of a comparator group of patients from the SMSreg, which the user may further specify using a few basic parameters. Thus, the Function Watch permits comparison of the patient's status with an expected/aimed for state. English translation of the Swedish text: *Main heading*: Välj kriterier för jämförelse: Administrativ nivå (Riket); Kön; Ålder; Duration; Behandling; Medel/Median; Max 2 år gammal referensdata. Uppdatera. Choose matching criteria: Administrative level (Country); Gender; Age; Duration; Treatment; Mean/Median; Maximum 2 years old reference data. Update. *Legend*: ≤2 år gamla data; >2 år gamla data; Referensgrupp. ≤2 years old data; >2 years old data; Reference group. *Text written inside the graph*: EDSS; MSSS; MSFC; MSIS-29-Fys; MSIS-29-Psyk; SDMT; FSMC; FSS; EQ5D; Arbetsförmåga; MS-koll; SF-36-1. EDSS; MSSS; MSFC; MSIS-29-Physical; MSIS-29-Psychological; SDMT; FSMC; FSS; EQ5D; Work capacity; MS-check; SF-36-1. *Explanations of the scales presented in a diagram* ‘*Functional watch*’: EDSS, Expanded Disability Status Scale; MSSS, Multiple Sclerosis Severity Scale; MSFC, Multiple Sclerosis Functional Composite; MSIS-29-Physical: Multiple Sclerosis Impact Scale – 29 – Physical part (20 questions); MSIS-29-Psychological: Multiple Sclerosis Impact Scale – 29 – Psychological part (nine questions); SDMT, Symbol Digit Modalities Test; FSMC, Fatigue Scale for Motor and Cognitive Functions; FSS, Fatigue Severity Scale; EQ5D, EuroQoL Group health questionnaire; Activity/Work capacity: Scale designed for the purpose of the SMS, evaluating limitations of activity/work capacity; MS-check: Multiple Sclerosis check-list based on Guy's scale; SF-36-1: First question from the SF-36 Health Survey.

The SMSreg interface effectively summarizes the key information needed to make decisions on key features of management, such as DMD prescription. In addition, SMSreg offers the possibility of comparing the patient in relation to other patients by assigning the MS severity score (MSSS) from the EDSS and disease duration. Recently, ‘the function watch’ diagram was introduced. It compares the patient across 12 functional scales (when available) to a group of reference patients that are matched according to preference (Fig.3). Thereby, the patient is compared to similar patients in the same database according to gender, age, disease duration, clinical course, and treatment strategy.

**Figure:**
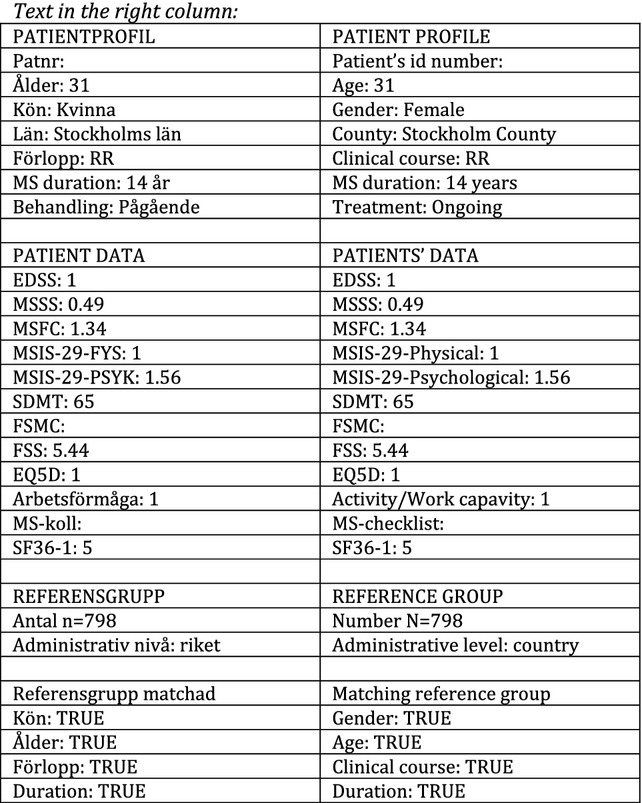


### Patient participation

**Figure 4:**
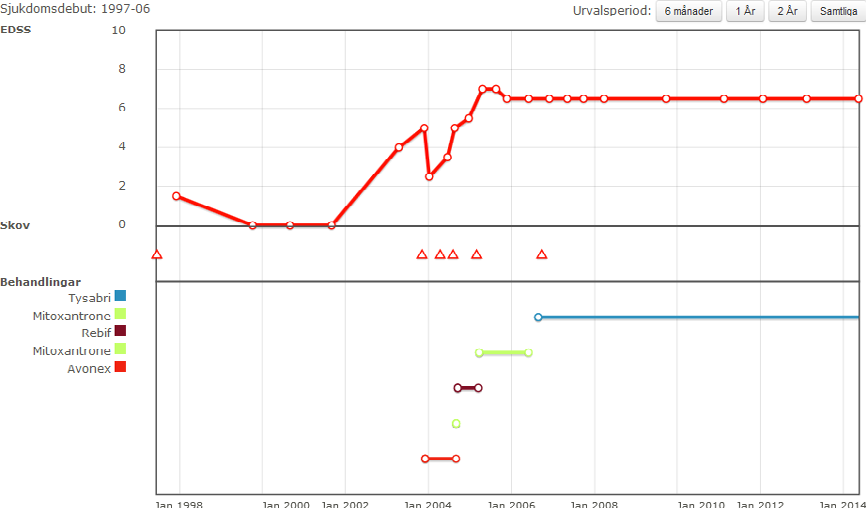
The interface of summarized SMSreg data on the patient. The patients can access the data by log-in from home at will. This interface represents an early version that will be developed further containing more variables. Translation of the Swedish text: *Main heading*: Sjukdomsdebut: 1997–2006; Urvalsperiod: 6 månader; 1 År; 2 År; Samtliga. Date of onset: 1997–2006; Select a period: 6 months; 1 year; 2 years; Whole period.

Important information on a patient's condition is assessed by patient-reported outcome questionnaires including the Multiple Sclerosis Impact Scale (MSIS-29) 3 and the EuroQuol 5D (EQ5D) 4. Some of these questionnaires can be completed online by patients, either from computers or tablets in the outpatient wards or from similar devices, including smartphones, from home after secure log-in. Recently, patients also have the possibility of logging onto the SMSreg and accessing a simplified version of the graphic interface used by clinicians (Fig.4). Whether this will develop into a useful and appreciated function of the SMSreg remains to be seen.

**Figure:**
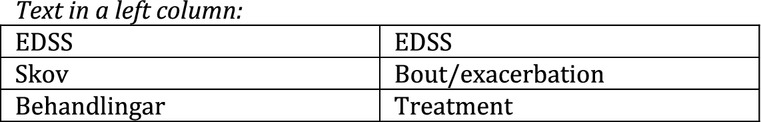


### Data access

A third functionality of most likely equal importance is the participating clinician's/center's access to their own data. For a long time, most data could be readily queried and then exported into Excel format for further analysis. As this may still be less user-friendly, we have developed a Visualization and Analysis Platform (VAP) that performs a number of queries and analyses in real time and presents them in graphical or table form 5. Currently, 20 such reports are available. Several parameters can be chosen, and a neurologist can choose to view data of his own patients or the department's patients.

### ‘Open comparisons’

**Figure 5:**
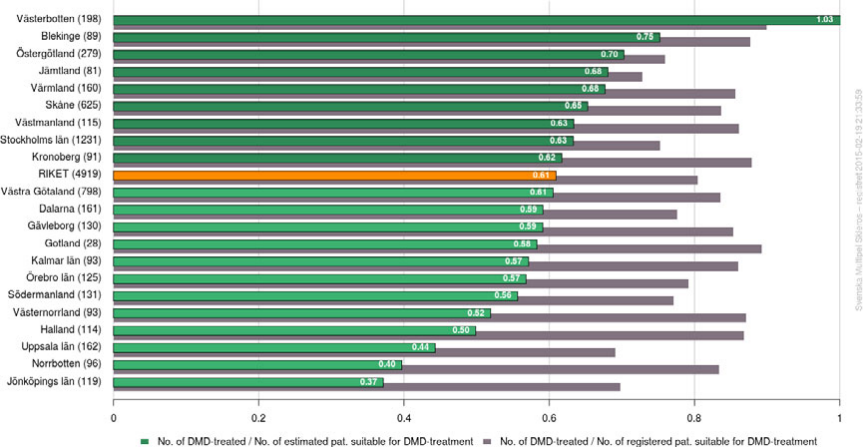
The proportion of early MS patients (RRMS with <15 years of duration) currently on disease-modifying drugs (DMDs) in Sweden (‘RIKET’) and in the 21 counties of Sweden. Gray bars show the proportion of patients on treatment in the registry, whereas green bars show the proportion of the estimated prevalent patient numbers in each county. Graphics are generated in real time from registry data using the Visualization and Analysis platform (VAP).

The organization of SMSreg conforms to the principle of openness. Therefore, 14 of the 20 VAP graphs/tables are public, although so far only with Swedish headings and legends are on http://msvap.demo.carmona.se. Thus, data is public from the county level up to national data, rendering information on departments and single physicians accessible only after authorized log-in. One of the graphs being publicly displayed on our homepage is shown (Fig.5).

## Research

To date, SMSreg has contributed data to over 100 scientific reports. The scope has ranged from epidemiology and genetics to neuroimmunology and treatment research. Some key publications to which SMSreg has contributed an important basis are the following.

### ‘Virtual placebo’

An expressed objective of SMSreg from its very start was to provide ‘real world evidence’ for the long-term effectiveness of DMDs. The concept behind this project was to use the Gothenburg natural course cohort as a provider of matched controls. In previous reports, this cohort was used to identify characteristics of the presenting MS attack that may serve as predictors for later MS disability. In 2013, Tedeholm et al. 6 reported that today's DMD-treated MS patients have less than half the risk of reaching secondary progression within 12 years of disease compared to matched controls from the Gothenburg cohort.

### Familial risks of MS

Westerlind et al. assessed the risk of MS among relatives to all patients with an MS diagnosis, either in SMSreg or in the national healthcare diagnosis registry in comparison with the risk among relatives of persons without MS. This was possible through the existence of a general registry of all parent–child relations in the Swedish population. Thus, Westerlind could assess the absolute and relative risks on practically all of the population, minimizing ascertainment bias 6. In a single paper, all first, second, and some third degree relations were assessed together with twins and relatives by adoption. Whereas crude risks were more or less as previously reported, Westerlind found that the relative risks were rather modest, for example with a relative risk of 7 for MS siblings 7.

#### MS genomewide association screens, the immunoChip, MS replication, and exome chip projects

In a series of papers, the International MS Genetics consortium has effectively increased the number of MS genetic susceptibility loci from 2 to over 200. SMSreg has contributed to this by being the basis for patient identification for patient DNA sampling and by contributing clinical data on the donators 8,9.

#### IMSE

Since the launch of Tysabri®(natalizumab, Biogen, Cambridge, MA, USA) in 2006, Swedish MS patients prescribed newly licensed DMDs have been asked to participate in an open-label follow-up study in which the SMSreg has served as an electronic clinical report form. This has enabled a variety of studies, as participating patients have also contributed DNA and serum samples and completed lifestyle questionnaires 10.

#### Genes and environment in MS

The ‘Epidemiology in MS’ (EIMS) project, an incident cohort of prospectively recruited patients with MS and matched population controls, has since its start in 2006 included 2805 patients with MS and 5674 controls (March 2015). Diagnostic details and clinical information on these patients are documented in the SMSreg. In 2008, 7500 patients in the SMSreg not yet recruited to EIMS or IMSE were invited to contribute DNA and plasma samples and to fill in a similar questionnaire in a project designated ‘Genes and environment in MS’ (GEMS). Eventually, over 5000 patients agreed to participate as well as close to 4000 matched population controls. The combined data sets of EIMS and GEMS have allowed the confirmation or primary identification of a number of environmental/lifestyle MS risk factors published in a series of reports 11–21. The combined access to both lifestyle and extensive genotyping available on patients recruited to the IMSE, EIMS, and GEMS studies offers a unique possibility to investigate whether genetic and environmental risk factors interact in predisposing to MS. So far, the most striking discovery of this class is that HLA genotype interacts with smoking habits by increasing the risk of MS in a fashion that renders persons carrying both risk factors at a significantly greater risk than the sum of the 2. This finding was recently confirmed in an independently collected material (to be published). The significance of this finding lies in the assumption that interaction is only possible when the two risk factors share an influence on the same disease mechanisms, thus providing pathogenetic clues.

### Anti-drug antibodies

Another example when the cross-analysis of different data sets available on patients in the SMSreg has allowed new discoveries is the identification of different HLA genotypes that increase the risk of antibodies to different interferon beta species 22 and that this risk is also influenced by smoking 23.

### Disease heterogeneity

The SMSreg has been used to identify patients lacking the MS-typical oligoclonal band (OCB) pattern seen on electrophoretic separation of immunoglobulin gamma (IgG). After a first report of a specific immunogenetic property among OCB-negative (OCB−) MS patients from SMSreg 24, a genetic difference between OCB− and OCB+ MS patients has now been confirmed and extended, with a major contribution of SMSreg patients 25,26. After confirming that OCB− patients differ also with regard to imaging properties 27,28, efforts are now ongoing to identify possible clinical traits of OCB− MS.

## Ongoing efforts

Given that over 9000 patients with MS from the SMSreg and almost as many controls from the IMSE, EIMS, and GEMS studies have been extensively genotyped; a wide array of studies are now being planned or are already ongoing to study the influence of genes and /or lifestyle on for instance disease course.

Another important current effort is to link SMSreg patient data to information from public registries, allowing for instance health economy studies.

For a complete list of SMSreg-related publications, please see http://www.neuroreg.se/Content/Files/Alla_publikationer_2003-2014_till_SMSreg_AR-2014_ver5_20141027.pdf.

## Future directions

### Expansion into other disease groups within neurology

The SMSreg presently contains information on over 16,000 patients of whom 14,500 are alive. Given the latest published prevalence figure of 17,000 patients with MS in 2008 29, this would equal 80% of population coverage. However, preliminary estimations indicate that the true prevalence is considerably higher today. Therefore, an increased coverage is essential for the SMSreg, both to quality assure MS care and to provide an optimal basis for research.

In recent years, SMSreg representatives have been active in promoting the design and implementation of similar registries for other diseases within neurology. Most importantly, there is now a start-up Swedish registry for Parkinson's disease of just over 1500 patients and a registry for myasthenia gravis with over 600 patients, some 25% of the prevalent cases. Registries for epilepsy, narcolepsy, motor neuron disease, inflammatory polyneuropathies, and severe vascular headache are also already launched. Together, these eight registries constitute the Swedish Neuro Registry (NEUROreg).

The aim of this expansion is most importantly to offer a similar tool for quality assuring neurology services and research for the most prevalent patient populations, but also to make routine use of SMSreg/NEUROreg a natural everyday practice for all neurologists, thereby increasing coverage and data density.

### International collaborations

For several research purposes, any given national registry and its patients may be too few for adequate statistical power. This is valid for many aspects of real world analysis, especially in the use and effectiveness of DMDs. As a consequence, representatives of leading MS registries and databases have formed collaborations to enable efficient pooling of data. These efforts need to overcome challenges of technical, ethical, legal, and political nature, but over long term, they are hoped to be of significance. If we ever are to work out principles of MS services, including DMD treatment to optimize the long-term quality of life for persons with MS, many MS neurologists need to come together in large-scale collaborations to collect clinical data.

## Conclusion

The SMSreg has shown that a voluntary MS registry can reach a promising degree of coverage by providing a useful tool for clinicians. Such a registry can be used to engage patients in their care as well as form a basis for research and inspire the implementation of similar registries for other chronic diseases. We propose that aspects of the SMSreg point to the future and that many of the properties of the SMSreg and other quality registries will eventually be inherent to most electronic medical record systems.
